# Supplementary material for: Comprehensive Genome-Wide Analysis of Histone Acetylation Genes in Roses and Expression Analyses in Response to Heat Stress
Source: Genes (Basel). 2022 May 30;13(6):980. doi: 10.3390/genes13060980 (PMC9222719; doi:10.3390/genes13060980)
Supplement: Supplementary file 1 [file genes-13-00980-s001.zip › genes-1708094-supplementary.pdf]

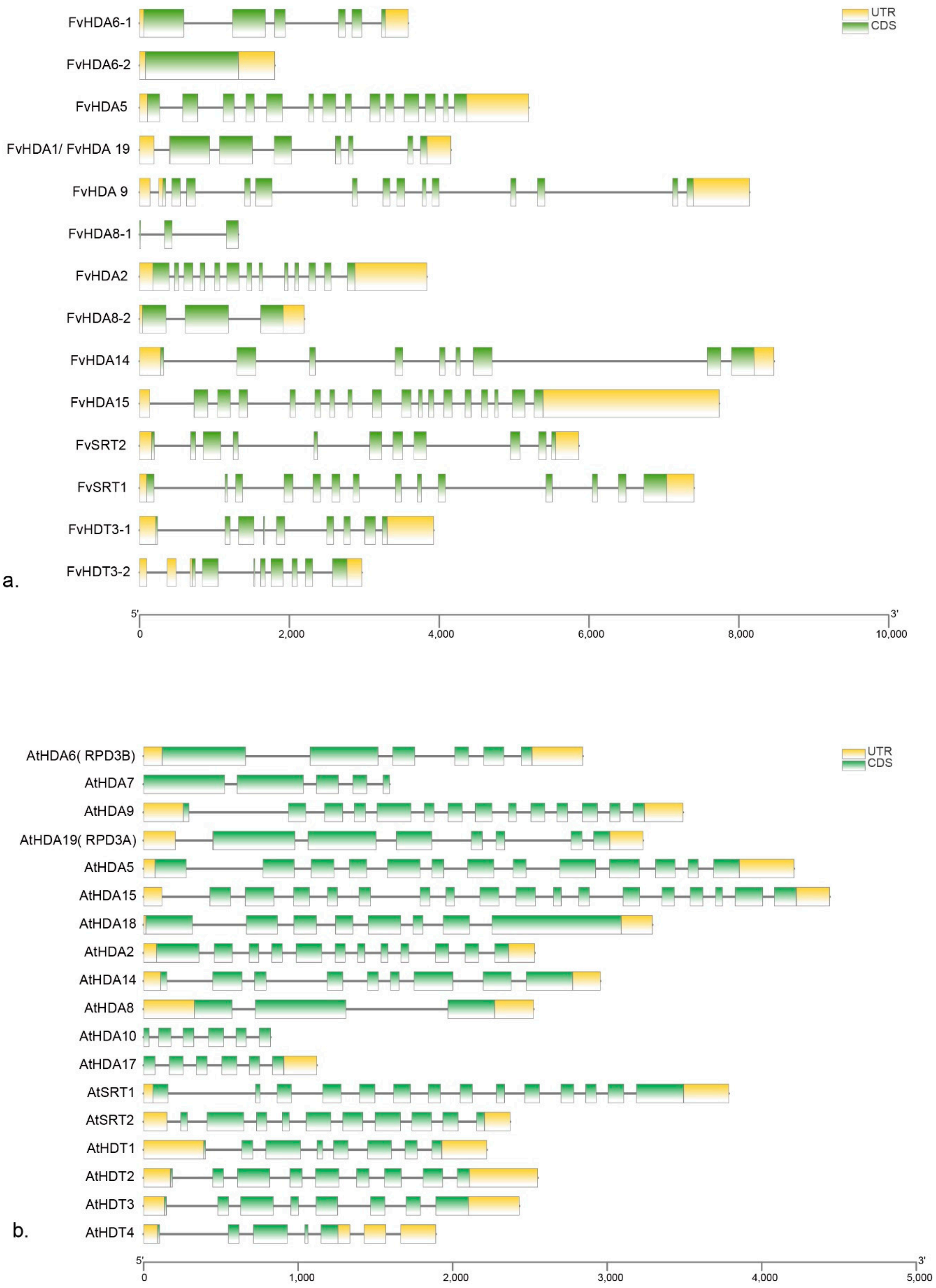

Figure S1. Structures of HDAC genes in *F. vesca* (**a**) and *A. thaliana* (**b**). UTR and CDS are indicated by yellow and green boxes, respectively. The lines indicate introns.

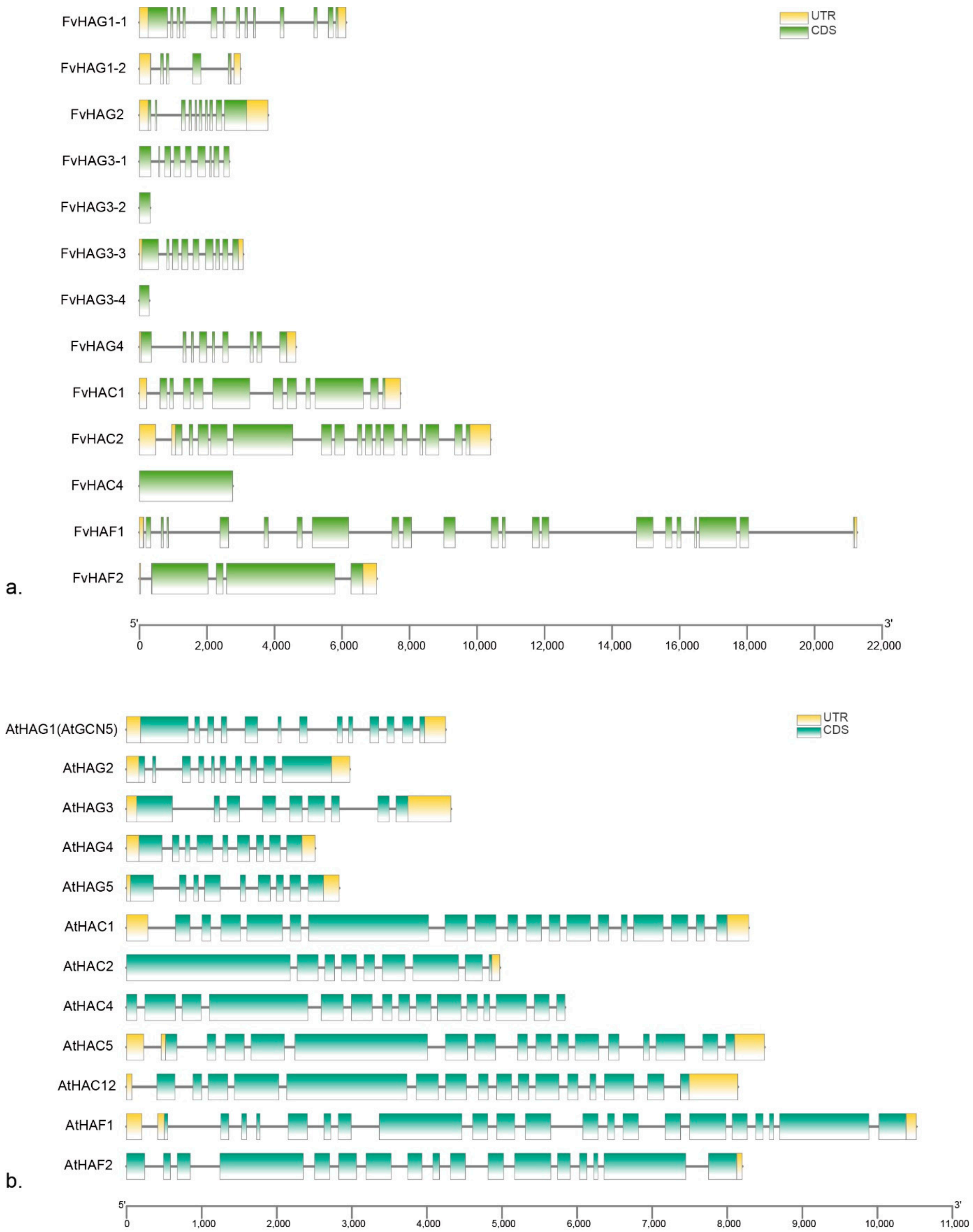

Figure S2. HAT genes structures of in *F. vesca* (**a**) and *A. thaliana* (**b**). UTR and CDS are indicated by yellow and green boxes, respectively. The lines indicate introns.

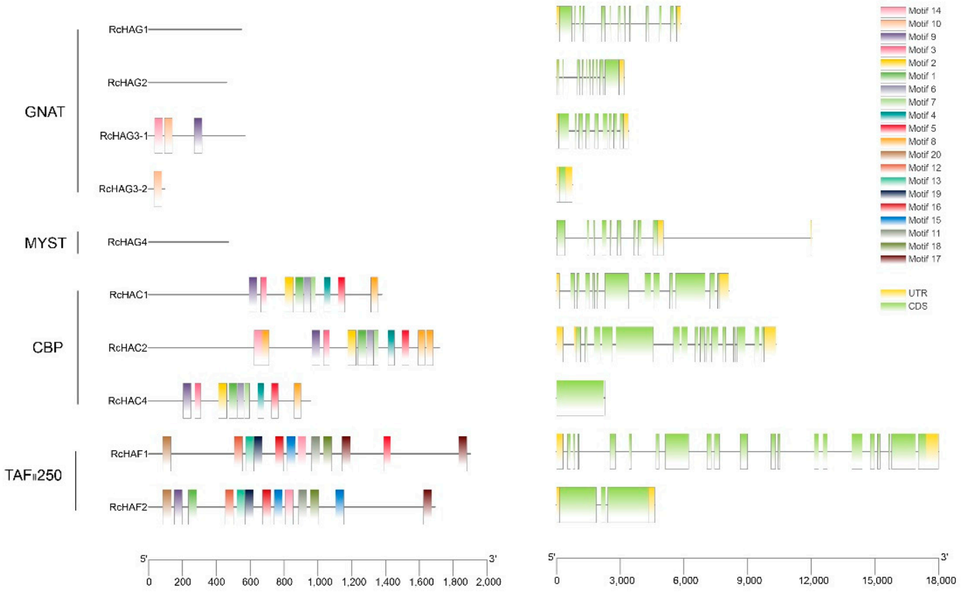

Figure S3. Conserved motifs and gene structures analysis of ten *R. chinensis* HATs. Motifs 1 to 20 are indicated by colored boxes. UTR and CDS are indicated by yellow and green boxes, respectively. The lines indicate introns.

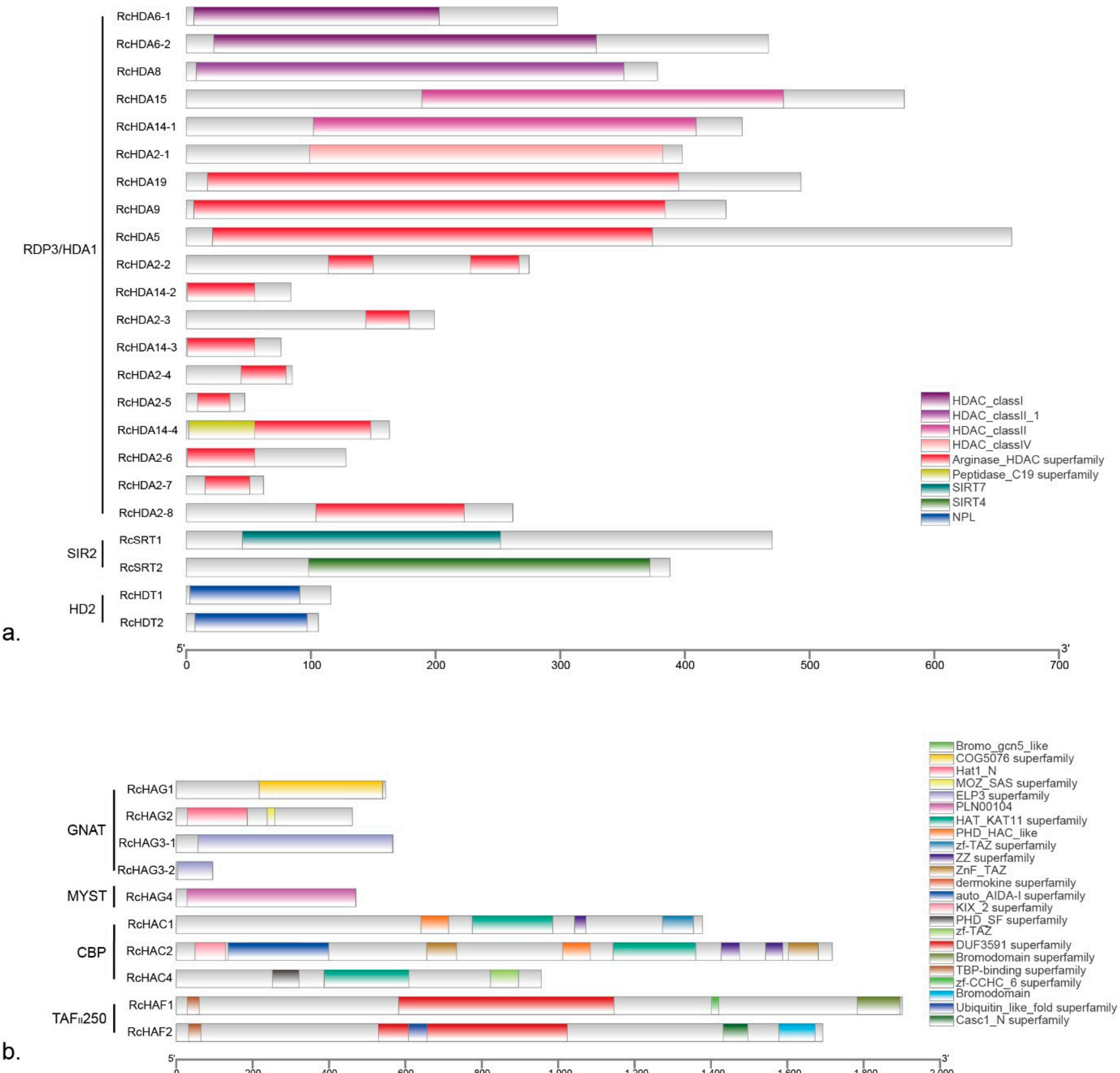

Figure S4. Conserved domains distribution in 23 HDAC proteins (**a**) and 10 HAT proteins (**b**) of *R. chinensis*. Domains are shown as colored boxes.

Table S1. HDAC and HAT genes in *A. thaliana*.

Gene subfamily	Gene ID	name
RPD3/HDA1	*AT5G63110.1*	*AtHDA6 (RPD3B)*
	AT5G35600.1	*AtHDA7*
	AT3G44680.1	*AtHDA9*
	AT4G38130.1	*AtHDA19 (RPD3A)*
	AT5G61060.2	*AtHDA5*
	AT3G18520.2	*AtHDA15*
	AT5G61070.1	*AtHDA18*
	AT5G26040.2	*AtHDA2*
	AT4G33470.1	*AtHDA14*
	AT1G08460.1	*AtHDA8*
	AT3G44660.1	*AtHDA10*
	AT3G44490.1	*AtHDA17*
SIR2	*AT5G55760.1*	*AtSRT1*
	AT5G09230.7	*AtSRT2*
HD2	*AT3G44750.1*	*AtHDT1*
	AT5G22650.1	*AtHDT2*
	AT5G03740.1	*AtHDT3*
	AT2G27840.1	*AtHDT4*
GNAT	*AT3G54610.1*	*AtHAG1 (AtGCN5)*
	AT5G56740.1	*AtHAG2*
	AT5G50320.1	*AtHAG3*
MYST	*AT5G64610.1*	*AtHAG4*
	AT5G09740.1	*AtHAG5*
CBP	*AT1G79000.2*	*AtHAC1*
	AT1G67220.1	*AtHAC2*
	AT1G55970.1	*AtHAC4*
	AT3G12980.1	*AtHAC5*
	AT1G16710.1	*AtHAC12*
$TAF_{II}250$	*AT1G32750.1*	*AtHAF1*
	AT3G19040.1	*AtHAF2*

Table S2. List of primers used in this study.

gene	Primer-F	Primer-R
RcHDA6-1	TTGCCTGACAACGACTACCAT	TATTGTCTTCCGCCTCCTCC
RcHDA6-2	TGTTGCTCGTTGCTGGTGCTATG	GTTGGCGGTGTTGTCTGGAAGG
RcHDA19	GCAGCAAGAAGAGTAAAGAAAGAGA	TCCATAGACCCAGAGCCCATA
RcHDA5	ATGTGCCTGTATAGAATTACGCTCG	TGGAATGTCACGCTCCTTGC
RcHDA15	GGGGAGGCGGAGATAAGTC	TACTTGAAACATCAGGCAGCG
RcHDA2-1	ACTCCGCCGCTCAAGAACTC	CGAAGAGTAAATCAACGGCACCT
RcHDA2-2	GTGGAGGAAGGCAATAGAGGTA	CTGAGAGATTTGGCATCGTTTAG
RcHDA2-5	CATCAATGTGGGAGGACTGG	ACCCTTGAGTATGTTTAACGGC
RcHDA8	TACTCAGCCTATTGTCTTCAT	GATAATCGCTCGTCCTCA
RcHDA14-2	TATATTCTTCCTTTCAACTCACCAA	GGAGTAAACTTCAAATCTAGACTAGCA
RcHDA14-3	CCTTTCAACTCACCAAGTAACAAT	CTAGACTAGCATAACAAGAACATATAA
RcSRT2	TGTCCAAACCTCAACCCAAGT	GGATCTGAATCAGGAGCCAACT
RcHDT1	ACCAAGGCAATGTGGTGCAT	CCCATTACGGATTGATAACCAGCA
RcHDT2	TCTTTCACAGGCCTGTCTGGA	TCCATGGAAGTGGACGCTCC
RcHAG1	CTGTACGGTGGGGCCTACTTC	CCCTGCTTCCTCCTCTCTCTT
RcHAG2	ATTTACCTGGTTTCTAGCAAA	GACTGTCAACAAGGGTCTCAC
RcHAG4	TAAACTTAGAATGGGTTCGATAGG	GCCGGAGTGAGGCTTGTT
RcHAC1	CAGCTGGCTTCGTACTTTGGT	GCTCATTCACATAGTCGTCCTCC
RcHAC4	CAATTTGACACCCTACGCC	TGCTGCTGCATGTCTTGTT
RcHAF1	CCAAATTACGTGATGCAGGAG	TTTTCAGAATCTAACGAGCCAAT
RcHAF2	AAGGGCTCGTAATGAGAAGGC	TTCATCACCCATAAGCAACCG
RcACTIN	TCAAGGATTGGTGGACTTCAGT	ACCAGAGAACAAGAATGCAAGC
RcGAPDH	GCTGGCAGGTATCCTTTCTG	GGCGACAATATCAGCCAAGT

Table S3. Genes encoding putative HDAC and HAT genes homologues in *R. chinensis*.

Subfamily	Gene ID	Name
RPD3/HDA1	*RchiOBHmChr3g0484871*	*RcHDA6-1*
	RchiOBHmChr7g0192801	*RcHDA6-2*
	RchiOBHmChr5g0048241	*RcHDA8*
	RchiOBHmChr7g0184671	*RcHDA15*
	RchiOBHmChr2g0121851	*RcHDA14-1*
	RchiOBHmChr6g0310741	*RcHDA2-1*
	RchiOBHmChr6g0281731	*RcHDA19*
	RchiOBHmChr7g0218461	*RcHDA9*
	RchiOBHmChr7g0183031	*RcHDA5*
	RchiOBHmChr4g0398391	*RcHDA2-2*
	RchiOBHmChr6g0252021	*RcHDA14-2*
	RchiOBHmChr4g0419371	*RcHDA2-3*
	RchiOBHmChr1g0346631	*RcHDA14-3*
	RchiOBHmChr5g0034671	*RcHDA2-4*
	RchiOBHmChr5g0034681	*RcHDA2-5*
	RchiOBHmChr6g0304611	*RcHDA14-4*
	RchiOBHmChr6g0269961	*RcHDA2-6*
	RchiOBHmChr6g0269981	*RcHDA2-7*
	RchiOBHmChr5g0028711	*RcHDA2-8*
SIR2	*RchiOBHmChr5g0053471*	*RcSRT1*
	RchiOBHmChr2g0122631	*RcSRT2*
HD2	*RchiOBHmChr5g0055321*	*RcHDT1*
	RchiOBHmChr3g0472041	*RcHDT2*
GNAT	*RchiOBHmChr6g0289651*	*RcHAG1*
	RchiOBHmChr2g0092581	*RcHAG2*
	RchiOBHmChr7g0191201	*RcHAG3-1*
	RchiOBHmChr5g0026811	*RcHAG3-2*
MYST	*RchiOBHmChr7g0240841*	*RcHAG4*
CBP	*RchiOBHmChr2g0163411*	*RcHAC1*
	RchiOBHmChr2g0148331	*RcHAC2*
	RchiOBHmChr3g0448221	*RcHAC4*
$TAF_{II}250$	*RchiOBHmChr3g0492481*	*RcHAF1*
	RchiOBHmChr5g0013481	*RcHAF2*

Table S4. Genes encoding putative HDAC and HAT genes homologues in *R. multiflora*.

Subfamily	Gene ID	Name
RPD3/HDA1	*Rmu_sc0003880.1_g000035.1*	*RmHDA14*
	Rmu_ssc0000289.1_g000024.1	*RmHDA15*
	Rmu_sc0004688.1_g000027.1	*RmHDA2*
	Rmu_ssc0000395.1_g000018.1	*RmHDA2*
	Rmu_sc0001408.1_g000004.1	*RmHDA8*
	Rmu_sc0002040.1_g000028.1	*RmHDA6*
	Rmu_sc0000014.1_g000006.1	*RmHDA5*
	Rmu_ssc0000126.1_g000063.1	*RmHDA19*
	Rmu_sc0001301.1_g000083.1	*RmHDA9*
	Rmu_sc0006898.1_g000006.1	*RmHDA14*
	Rmu_sc0010612.1_g000002.1	*RmHDA14*
	Rmu_sc0002351.1_g000007.1	*RmHDA14*
	Rmu_sc0003880.1_g000030.1	*RmHDA2*
	Rmu_sc0003720.1_g000019.1	*RmHDA2*
	Rmu_sc0003184.1_g000002.1	*RmHDA14*
	Rmu_sc0006653.1_g000010.1	*RmHDA6*
	Rmu_sc0002194.1_g000035.1	*RmHDA14*
	Rmu_sc0021556.1_g000004.1	*RmHDA9*
SIR2	*Rmu_sc0005966.1_g000015.1*	*RmSRT1*
	Rmu_sc0003133.1_g000010.1	*RmSRT2*
HD2	*Rmu_sc0003641.1_g000018.1*	*RmHDT1*
	Rmu_co8477099.1_g000001.1	*RmHDT1*
	Rmu_sc0002532.1_g000010.1	*RmHDT1*
	Rmu_sc0001665.1_g000047.1	*RmHDT1*
	Rmu_sc0011151.1_g000006.1	*RmHDT1*
	Rmu_sc0012467.1_g000004.1	*RmHDT2*
GNAT	*Rmu_sc0005376.1_g000013.1*	*RmHAG1*
	Rmu_co8234453.1_g000001.1	*RmHAG3-1*
	Rmu_sc0019130.1_g000001.1	*RmHAG3-2*
	Rmu_sc0003674.1_g000014.1	*RmHAG3-3*
	Rmu_ssc0000172.1_g000023.1	*RmHAG3-4*
	Rmu_sc0016007.1_g000009.1	*RmHAG3-5*
MYST	*Rmu_sc0002313.1_g000023.1*	*RmHAG4*
	Rmu_co8495669.1_g000001.1	*RmHAG5-1*
	Rmu_sc0004821.1_g000016.1	*RmHAG5-2*
CBP	*Rmu_ssc0000231.1_g000004.1*	*RmHAC1*
	Rmu_sc0005969.1_g000037.1	*RmHAC2*
	Rmu_sc0005969.1_g000044.1	*RmHAC4*
$TAF_{II}250$	*Rmu_sc0003458.1_g000019.1*	*RmHAF1*
	Rmu_sc0020049.1_g000002.1	*RmHAF2-1*
	Rmu_sc0000971.1_g000001.1	*RmHAF2-2*

Table S5. Gene encoding putative HDAC and HAT genes homologues in *F. vesca*.

Subfamily	Gene ID	Gene ID in old nomenclature	Name
RPD3/HDA1	*FvH4_5g06060.t1*	*gene09844*	*FvHDA6-1*
	FvH4_1g25790.t1	*gene12349*	*FvHDA6-2*
	FvH4_5g13910.t1	*gene37149*	*FvHDA5*
	FvH4_2g17240.t1	*gene17127*	*FvHDA1/ FvHDA 19*
	FvH4_5g27310.t1	*gene29263*	*FvHDA 9*
	FvH4_3g26570.t1	*gene40220*	*FvHDA8-1*
	FvH4_2g32010.t1	*gene35931*	*FvHDA2*
	FvH4_3g26560.t1	*gene27053*	*FvHDA8-2*
	FvH4_1g24510.t1	*gene16203*	*FvHDA14*
	FvH4_5g12660.t1	*gene26215*	*FvHDA15*
SIR2	*FvH4_1g24990.t1*	*gene16117*	*FvSRT2*
	FvH4_3g29170.t1	*gene15261*	*FvSRT1*
HD2	*FvH4_6g17790.t1*	*gene30136*	*FvHDT3-1*
	FvH4_3g29940.t1	*gene14356*	*FvHDT3-2*
GNAT	*FvH4_2g22590.t1*	*gene35776*	*FvHAG1*
	FvH4_1g06810.t1	*gene11375*	*FvHAG2*
	FvH4_4g08670.t1	*gene20524*	*FvHAG3-1*
	FvH4_5g07450.t1	*gene41363*	*FvHAG3-2*
	FvH4_5g07410.t1	*gene41358*	*FvHAG3-3*
	FvH4_5g07340.t1	*gene09922*	*FvHAG3-4*
	FvH4_3g25041.t1	*gene21768*	*FvHAG3-5*
MYST	*FvH4_5g38850.t1*	*gene02241*	*FvHAG4*
CBP	*FvH4_6g45470.t1*	*gene10338*	*FvHAC1*
	FvH4_6g36320.t1	*gene37806*	*FvHAC2*
	FvH4_6g00740.t1	*gene16783*	*FvHAC4*
$TAF_{II}250$	*FvH4_1g29900.t1*	*gene30351*	*FvHAF1*
	FvH4_3g05980.t1	*gene30562*	*FvHAF2*

Table S6. Genes encoding putative HDAC and HAT genes homologues in *R. rugosa*.

Subfamily	Gene ID	name
RPD3/HDA1	*evm.model.Chr5.3090*	*RrHDA8*
	evm.model.Chr6.3259	*RrHDA14*
	evm.model.Chr7.515	*RrHDA5*
	evm.model.Chr7.659	*RrHDA15*
	evm.model.Chr2.3097	*RrHDA19*
	evm.model.Chr7.1281	*RrHDA6*
	evm.model.Chr7.3446	*RrHDA9-1*
	evm.model.Chr7.3445	*RrHDA9-2*
	evm.model.Chr2.220	*RrHDA2-1*
	evm.model.Chr5.620	*RrHDA2-2*
SIR2	*evm.model.Chr5.2852*	*RrSRT1*
HD2	*evm.model.Chr5.2728*	*RrHDT1*
GANT	*evm.model.Chr2.2236*	*RrHAG1-1*
	evm.model.Chr2.2382	*RrHAG1-2*
	evm.model.Chr6.759	*RrHAG2*
	evm.model.Chr7.1155	*RrHAG3-1*
	evm.model.Chr5.413	*RrHAG3-2*
MYST	*evm.model.Chr7.5663*	*RrHAG4*
CBP	*evm.model.Chr6.4885*	*RrHAC1*
	evm.model.Chr6.6060	*RrHAC2*
	evm.model.Chr4.83	*RrHAC4*
$TAF_{II}250$	*evm.model.Chr3.1433*	*RrHAF1*
	evm.model.Chr5.5920	*RrHAF2*

Table S7. The FPKM values of HDAC and HAT genes in two *Rosa* species (*R. chinensis* 'OB' , *R. wichurana* 'Rw') and one modern rose cultivar (*R. hyb*rida 'Yesterday' 'Ry').

Gene ID	Ro	Ste	Le	Sta	Ca	FB_GP	FB_CP	FB_PP	OF_PP	Rw_root	Rw_stem	Rw_leaf	Rw_bud	Ry_root	Ry_stem	Ry_leaf	Ry_bud
RcHDA6-1	0.0000	0.0000	0.0000	0.0000	0.0000	0.0000	0.0000	0.0000	0.0000	0.0000	0.0000	0.0000	0.0000	0.0000	0.0000	0.0000	0.0000
RcHDA6-2	68.1931	33.9198	28.3161	33.0634	40.4769	31.5418	28.0425	27.9431	28.2591	22.2996	38.1760	5.2150	21.8344	24.4309	29.8293	4.5677	4.0711
RcHDA9	15.7086	11.3277	12.9646	15.2004	16.3715	16.8983	14.3275	16.0481	21.2195	6.8115	7.2895	7.5479	9.5202	6.7564	5.9611	9.9143	3.0289
RcHDA19	33.1515	31.0477	22.9788	26.3391	45.6942	68.9842	52.4317	32.1443	28.7274	18.7022	33.0654	9.9082	56.1723	40.4236	31.7014	11.2303	11.7476
RcHDA5	5.3330	11.2574	7.4007	7.3819	11.6621	10.4497	8.8706	9.8802	9.5644	7.0039	4.5977	4.5117	13.6431	11.6056	4.9170	4.1314	1.1618
RcHDA15	14.3632	11.7600	12.1302	8.2263	16.9770	23.8132	16.8517	14.5127	14.0623	7.8336	4.9695	5.7866	13.9829	7.3094	2.7549	8.0570	3.4514
RcHDA2-1	7.0014	6.2643	7.1192	3.1700	7.8830	6.6625	5.8482	4.3865	2.5810	3.8068	3.8776	3.6504	3.5494	4.7202	5.0939	3.5281	0.8834
RcHDA2-2	0.0000	0.0000	0.0000	0.3754	0.0000	0.0641	0.0000	0.0342	0.0247	0.0000	0.0000	0.6927	1.0737	0.0000	0.0000	0.0000	0.0000
RcHDA2-3	0.0000	0.0000	0.0000	0.3518	0.1327	0.0235	0.1325	0.1446	0.7732	0.0000	0.1188	0.0000	0.0000	0.0000	0.0000	0.0000	0.0000
RcHDA2-4	0.0909	0.0000	0.0000	0.0000	0.0000	0.0195	0.0578	0.0104	0.0845	0.0000	0.0000	0.0000	0.0000	0.0000	0.0000	0.0000	0.0000
RcHDA2-5	0.2254	0.0000	0.0000	0.0000	0.0000	0.0000	0.0000	0.0319	0.0516	0.0000	0.0000	0.0000	0.0000	0.0000	0.0000	0.0000	0.0000
RcHDA2-6	0.0000	0.0000	0.0000	0.0000	0.0000	0.0000	0.0000	0.0000	0.0000	0.0000	0.0000	0.0000	0.0000	0.0000	0.0000	0.0000	0.0000
RcHDA2-7	0.0000	0.0000	0.0000	0.0000	0.0000	0.0000	0.0000	0.0000	0.0000	0.0000	0.0000	0.0000	0.0000	0.0000	0.0000	0.0000	0.0000
RcHDA2-8	0.0000	0.0000	0.0000	0.0000	0.0000	0.0000	0.0000	0.0000	0.0000	0.0000	0.0000	0.0000	0.0000	0.0000	0.0000	0.0000	0.0000
RcHDA8	8.8498	12.3024	12.6038	4.5005	14.1289	10.8250	9.3778	5.7932	6.0205	4.6929	5.1043	4.2204	6.3255	6.9875	8.5854	8.7573	2.6562
RcHDA14-1	1.5716	1.8422	35.7098	0.6125	3.1743	3.2659	2.2479	1.9753	2.1350	1.0073	3.8203	33.9351	3.8945	0.2820	1.8873	19.6914	0.5048
RcHDA14-2	0.0000	0.0000	0.0011	0.0000	0.0000	0.0000	0.0000	0.0000	0.0000	0.0000	0.0000	0.0000	0.0000	0.0000	0.0000	0.0000	0.0000
RcHDA14-3	0.0000	0.0000	0.0012	0.0000	0.0000	0.0081	0.0033	0.0000	0.1842	0.0000	0.0000	0.0000	0.0000	0.0000	0.0000	0.0000	0.0000
RcHDA14-4	0.0000	0.0000	0.0000	0.0000	0.0000	0.0000	0.0000	0.0000	0.0000	0.0000	0.0000	0.0000	0.0000	0.0000	0.0000	0.0000	0.0000
RcSRT1	1.1230	2.5648	2.0542	2.0202	3.8120	6.6595	5.1210	3.4164	2.3537	1.2310	0.8315	1.7354	3.7786	1.3525	1.1302	2.9670	2.1712
RcSRT2	1.2859	2.0135	7.6563	6.6269	5.6303	6.6596	6.5398	13.2916	61.7715	2.7285	3.2299	9.0707	5.2049	1.9986	1.2590	10.6192	1.7019
RcHDT1	55.7067	39.4383	16.8726	9.8568	48.1027	75.9806	61.3818	23.1117	26.4237	19.9234	35.8015	8.0544	72.1256	32.1315	40.6580	12.0249	24.2593
RcHDT2	19.7365	50.8300	25.1223	37.2040	62.3613	69.4529	53.5825	50.3504	24.7683	9.2588	16.3931	18.8225	37.8389	13.3724	9.4953	27.7609	18.0468
RcHAG1	40.3758	18.8353	17.0497	18.3081	30.4076	28.7988	20.5563	15.5778	11.0712	13.5020	13.4019	3.7109	16.1484	12.7087	12.7984	5.0060	3.0419
RcHAG2	8.4054	13.1040	7.0987	4.3462	13.9784	17.9579	7.6617	5.5181	6.1029	8.6889	17.3268	2.3689	41.3577	14.0490	12.6186	4.7209	9.8482
RcHAG3-1	33.3840	12.5539	9.6280	6.4470	19.3447	45.0484	74.8625	33.8450	6.4529	10.4433	17.9833	2.8275	15.1442	12.1900	19.6265	1.3821	2.5073
RcHAG3-2	4.1221	3.4514	2.3028	0.8712	3.4352	2.7092	1.8352	0.8752	0.2171	0.0000	0.0000	0.0000	0.0000	0.0000	0.0000	0.0000	0.0000
RcHAG4	63.4138	29.7937	32.3686	31.2981	40.0903	45.1754	33.8188	33.6589	37.8126	17.2331	20.4130	15.0573	21.2715	22.3165	19.3228	14.1528	6.6573
RcHAC1	2.0300	0.9935	5.2661	3.3711	2.8370	5.8198	3.6232	1.7109	1.0468	0.9230	0.5309	0.1433	1.1033	0.9413	0.3669	0.0728	0.0969
RcHAC2	25.7755	16.6003	21.1333	16.9286	21.5600	19.7172	17.2877	18.1671	18.9509	11.0568	5.9854	1.3858	6.5107	13.4538	5.7406	0.8892	0.4081
RcHAC4	0.0000	0.0000	0.0000	0.1361	0.0726	0.9516	0.8626	0.4982	0.1796	0.0369	0.0000	0.0000	0.0000	0.0855	0.0353	0.0161	0.0000
RcHAF1	8.8430	7.1761	5.5243	10.3158	8.7373	9.3527	7.4391	9.6643	8.1126	5.3676	4.9543	0.4068	3.7509	6.3515	5.2237	0.1956	0.2868
RcHAF2	1.7758	2.0367	2.0384	1.7070	3.2219	3.9685	3.5418	3.4146	2.4542	1.1152	0.8675	0.0282	0.5996	1.0679	0.5825	0.0000	0.0000

Organs from R. chinensis 'Old blush' are labeled as Ro: roots; Ste: stems; Le: leafs; Sta: stamens; Ca: carpels; FB_GP: green petals in flower buds; FB_CP: petals changing colors in flower buds; FB_PP: pink petals ; and OF_PP: pink petals in open flowers. Organs from *R. wichurana* are labelled with 'Rw' (ex. Rw_root , etc.) , and those from *R. hyb*rida 'Yesterday' are labelled with 'Ry' (ex. Ry_root, etc.)

Table S8. The expression values of selected HDAC and HAT genes in 'CL' roses grown under control (25°C) and high temperature (35°C) conditions.

Gene ID	25Se	25Pt	25St	25Pi	25Le	35Se	35Pt	35St	35Pi	35Le
RcHDA6-1	4.8506±0.3496 d	4.0044±0.4806 de	2.5522±1.0698 e	14.1843±5.4867 b	18.1732±2.5814 a	1.8529±0.1237 ef	2.1873±0.0757 e	13.4190±1.8155 c	1.2006±0.3398 ef	0.5211±0.1317 f
RcHDA6-2	0.6086±0.1559 d	0.8326±0.1681 c	1.2235±0.1733 b	0.8752±0.2481 c	0.3556±0.0370 e	2.0772±0.3620 a	0.8983±0.2292 c	0.4692±0.0602 e	0.4051±0.0829 e	1.2411±0.2629 b
RcHDA19	8.8756±1.7906 b	3.4863±0.2931 e	2.8117±0.6788 e	2.1002±0.4115 f	12.4680±1.3201 a	5.3915±0.3534 c	4.0827±0.9299 d	3.0453±0.2521 e	1.1409±0.0779 g	4.5421±1.1014 cd
RcHDA5	0.5122±0.1167 f	0.9359±0.1783 d	0.7973±0.0371 e	1.7458±0.2067 b	0.3883±0.1425 f	1.9893±0.1648 a	0.4993±0.0307 f	1.2431±0.3989 c	0.9272±0.2636 d	0.7287±0.1170 e
RcHDA15	8.2781±1.8727 a	2.4296±0.3994 c	0.2769±0.0499 e	0.3497±0.0737 e	9.0287±0.1675 a	4.3087±1.3349 b	0.4998±0.0712 de	0.9688±0.2704 d	0.2576±0.0601 e	1.0343±0.2594 d
RcHDA2-1	20.0741±4.3331 b	0.9844±0.1826 de	0.6382±0.0851 de	3.2011±0.2953 d	28.0703±9.6093 a	15.3868±2.5633 c	1.3153±0.1641 de	1.9210±0.3392 de	0.5115±0.0853 e	2.7652±0.5453 de
RcHDA2-2	7.1299±1.9705 a	0.2199±0.0219 e	0.2897±0.1016 e	1.0555±0.2472 d	5.2490±0.7321 b	4.2725±0.4489 c	0.3270±0.0202 e	0.7861±0.1833 de	0.0570±0.0110 e	0.8395±0.1067 de
RcHDA2-5	2.1915±0.1662 d	0.5711±0.0445 ef	2.2329±0.6074 d	5.9364±2.0183 b	8.5245±1.7927 a	1.0480±0.0369 e	0.9097±0.0732 e	3.1186±0.2843 c	0.5502±0.0079 ef	0.2158±0.0330 f
RcHDA8	2.1739±0.2603 de	1.7529±0.1744 e	4.1586±0.4008 c	9.0505±2.5483 a	4.8722±1.3556 b	1.0716±0.0998 ef	2.1070±0.1591 de	4.3894±2.0469 bc	2.7560±0.2036 d	0.6352±0.1090 f
RcHDA14-2	30.3796±3.9640 a	1.8330±0.0958 e	0.4198±0.0925 e	4.6780±1.3161 d	18.1518±1.7680 c	19.8382±3.8849 b	1.7624±0.2504 e	3.7971±2.2511 d	0.4414±0.0149 e	3.9057±0.1973 d
RcHDA14-3	2.2413±0.0396 a	0.0579±0.0006 f	0.0570±0.0184 f	0.4044±0.1950 d	1.2027±0.2751 c	1.3299±0.2324 b	0.1038±0.0370 f	0.2148±0.1357 e	0.0196±0.0027 f	0.2442±0.1508 e
RcSTR2	2.3103±0.2727 b	0.8531±0.0663 d	0.1770±0.0383 ef	0.1115±0.0227 f	11.0094±0.7852 a	1.4734±0.1468 c	0.6476±0.1798 d	0.3609±0.0382 e	0.0853±0.0176 f	0.8643±0.1583 d
RcHDT1	19.0941±1.7986 b	6.1130±1.5702 e	6.9274±1.6633 e	5.2706±0.4472 ef	32.8228±8.2153 a	12.9824±2.6456 c	6.9875±1.1008 e	10.2001±2.4904 d	3.6447±0.8866 f	14.5149±0.8345 c
RcHDT2	3.1477±0.4337 f	14.5394±2.1136 b	26.2947±4.0051 a	12.3364±0.9434 c	10.4573±2.1982 d	1.0000±0.1139 g	8.3614±1.2254 e	10.1392±1.5520 d	12.2903±0.8917 c	0.5743±0.1000 g
RcHAG1	280.4564±73.7220 c	374.4544±80.5126 b	120.5811±22.7445 e	58.3551±10.3943 f	1.0000±0.2309 g	183.5061±52.0257 d	164.4738±13.9708 de	429.7945±124.1338 a	103.1349±23.5513 e	396.8948±18.2811 b
RcHAG2	212.4227±30.9777 f	567.5547±169.4520 c	105.7994±28.8740 g	28.5742±3.9418 h	1.7770±0.4721 h	903.9821±89.8152 b	275.5408±37.6193 e	195.0845±64.7209 f	339.3324±19.4268 d	1211.4075±49.1641 a
RcHAG4	826.2873±27.4253 g	2645.1158±178.6772 d	677.9957±133.0009 g	318.4642±38.4116 h	248.1073±26.6545 h	4504.2930±332.9750 a	1527.1612±269.3548 e	1270.5757±502.8011 f	3666.4884±143.3607 b	3317.7079±235.6567 c
RcHAC4	191.5793±10.9531 a	21.0705±3.8870 f	7.0724±1.4421 g	19.0281±2.4499 fg	12.1759±1.5031 g	162.7796±16.7516 b	135.2246±7.0768 c	107.7987±25.1440 d	15.2267±2.6003 fg	96.6832±2.8881 e
RcHAF2	774.3281±79.4892 a	308.7874±3.3538 c	101.4640±23.4234 f	71.2960±10.7882 g	15.2515±1.8190 h	259.1747±40.4402 d	260.5073±19.1848 d	241.8952±22.5972 de	417.2425±11.6523 b	238.5693±5.1324 e

Means with different letters (a, b, c,…) indicate statistically significantly different at $P < 0.05$, Single Factor ANOVA along with Least Significant Difference test).
